# Stepwise Embryonic Toxicity of Silver Nanoparticles on *Oryzias latipes*

**DOI:** 10.1155/2013/494671

**Published:** 2013-07-30

**Authors:** Jae-Gu Cho, Kyung-Tae Kim, Tae-Kwon Ryu, Jae-woo Lee, Ji-Eun Kim, Jungkon Kim, Byoung-Cheun Lee, Eun-Hye Jo, Junheon Yoon, Ig-chun Eom, Kyunghee Choi, Pilje Kim

**Affiliations:** Risk Assessment Division, National Institute of Environmental Research, Kyungseo-Dong, Seo-gu, Incheon 404-170, Republic of Korea

## Abstract

The developmental toxicity of silver nanoparticles (AgNPs) was investigated following exposure of *Oryzias latipes* (medaka) embryos to 0.1−1 mg/L of homogeneously dispersed AgNPs for 14 days. During this period, developmental endpoints, including lethality, heart rate, and hatching rate, were evaluated by microscopy for different stages of medaka embryonic development. To compare toxic sensitivity, acute adult toxicity was assessed. There was no difference in acute lethal toxicity between embryo and adult medaka. Interestingly, we found that the increase in stepwise toxicity was dependent on the developmental stage of the embryo. Lethal embryonic toxicity increased from exposure days 1 to 3 and exposure days 5 to 8, whereas there was no change from exposure days 3 to 5. In addition, 7 d exposure to 0.8 mg/L AgNPs resulted in significant heart beat retardation in medaka embryos. AgNPs also caused a dose-dependent decrease in the hatching rate and body length of larvae. These results indicate that AgNP exposure causes severe developmental toxicity to medaka embryos and that toxicity levels are enhanced at certain developmental stages, which should be taken into consideration in assessments of metallic NPs toxicity to embryos.

## 1. Introduction

Manufactured nanoparticles (NPs) are particles of 100 nm diameter or less. The applications of NPs have extended in recent years to areas such as medicine, pharmacology, electronic engineering, magnetic fields and semiconductors, biotechnology, materials and process development, energy, and environmental remediation [1–6]. It is estimated that nanotechnology will represent $1.5 trillion worth of the global market by 2015 [7]. While such technology provides numerous benefits, its potential toxic effects on the physiology of humans and animals have led to mounting concerns regarding potential environmental and human health risks associated with exposure to nanomaterials.

Silver nanoparticles (AgNPs) have emerged as an important class of nanomaterials and are currently used in a wide range of industrial applications and healthcare products with their antimicrobial effects [8, 9]. With the broad range of nanoparticle use, the potential severity of AgNP contamination in the aquatic environment has begun to be acknowledged. AgNPs have a large surface area-to-volume ratio, which means they potentially provide an efficient means of delivering toxicity. A large number of studies have provided strong support for the hypothesis that Ag ions have a more toxic contribution than AgNP [7, 10–14]. However, similar to other NPs, little is yet known about the specific mechanisms and modes of actions regarding AgNP toxicity.

The extensive application of AgNPs might eventually lead to their release into the environment [15], causing toxic effects on aquatic organisms. Thus, many studies have been undertaken to examine AgNP toxicity to aquatic organisms, including algae [16], cladocerans [17], and small fish [14, 18, 19]. However, only a few studies have focused on embryonic toxicity [7, 11, 20] or differences in sensitivity to AgNP between embryos and adult fish. In this study, we investigated the lethal and embryonic toxicity of AgNP dispersion (hydrodynamic size: 36.8–55.3 nm) in embryo and adult medaka (*Oryzias latipes*). This fish species has been used as a model organism in a wide variety of research fields, particularly in embryonic development, because individuals are transparent and easily observed under a microscope.

## 2. Material and Methods

### 2.1. Determination and Characterization of AgNPs

AgNP colloid capped with citrate (CAS 7440-22-4, silver nanoparticles) was purchased from ABC NanoTech Co., Ltd. (Daejeon, Korea). To obtain the UV-Vis spectrum of AgNPs, the original colloid was diluted in deionized water (DW) or culture water at 10 mg/L, and scanned from 200 to 600 nm using a spectrophotometer (Ultrospec 2000; Pharmacia Biotech Ltd., UK). The zeta potentials of AgNP dispersions were measured using dynamic light scattering method (ELS-PT, Otsuka Electronics, Japan) to check the pH-dependent variation in AgNP. The hydrodynamic sizes of AgNP were measured using a dynamic light scattering (DLS) method (ELS-PT; Otsuka Electronics), and particle characteristics were measured by transmission electron microscopy (TEM, Tecnai 20; Philips, Netherlands).

### 2.2. Culture and Maintenance of **O. latipes**

The medaka used in this study was cultured by the National Institute of Environmental Research (Incheon, Korea). The culture and maintenance of *O. latipes *were performed at 23 ± 1°C in 50-L aquariums containing 40 L of culture water prepared following the Organisation for Economic Co-operation and Development (OECD) guidelines [21]. Light conditions were controlled using a 16 : 8 h light : dark photoperiod. Fish were fed with *Artemia salina* nauplii once a day. Eggs were pooled and washed in culture water and then screened under a stereo dissecting microscope (Stemi SV11; Zeiss Co., Germany). The embryonic developmental stage was determined as reported by a previous study [22]. Fertilized eggs at developmental stages 10~11 (blastula) were used in this study. 

### 2.3. AgNP Exposure

An acute adult lethality test was conducted as outlined by OECD Technical Guide 203 [21]. Seven adults (~4 months old) were placed in a 3-L beaker containing 2 L of AgNP test solution. Abnormal behavior and lethality were checked and recorded daily after exposure. Basic water chemistry, such as dissolved oxygen, pH, temperature, and conductivity, was measured and recorded before and after the renewal of the medium.

A short-term test of AgNP toxicity on embryos and sac-fry stages (larvae) was conducted with fertilized eggs for 14 d, as outlined in OECD Technical Guide 212 [23]. Thirty fertilized eggs were randomly divided equally into a 6-well polystyrene plate (i.e., 10 eggs per well) containing 3 mL of AgNP solution for each treatment. Test solutions were renewed every second day. The heart rate of embryos was measured at day 3, 5, and 7. Survival, phenotypic deformities, and hatchability were monitored daily with microscopy (Stemi SV11; Zeiss Co., Germany) until day 14. Embryos that did not hatch within 14 days were defined as dead. After hatching, the heart beat and length of larvae were determined. Hatchability was calculated from the sum of dead and unhatched individuals. Larvae were reported as dead if the heart stopped beating and/or did not respond when gently touched. 

Exposure concentration was determined as a range of 0.1, 0.25, 0.5, 0.75, and 1.0 mg/L for both the adult 96 h acute toxicity tests and embryo toxicity tests.

### 2.4. Statistical Analysis

The median lethal concentrations (LC50) and associated 95% confidence intervals (CIs) were calculated by Probit analysis (Probit analysis program, version 1.5.; WEST, Cheyenne, WY, USA). The significant difference of LC50 value was considered to be present if the CI. One-way analyses of variance (ANOVA) followed by Dunnett's test as a post hoc test were performed for heart rate, hatching rate, and body length data. The results were reported as mean ± standard deviation.

## 3. Results and Discussion

**Figure 1:**
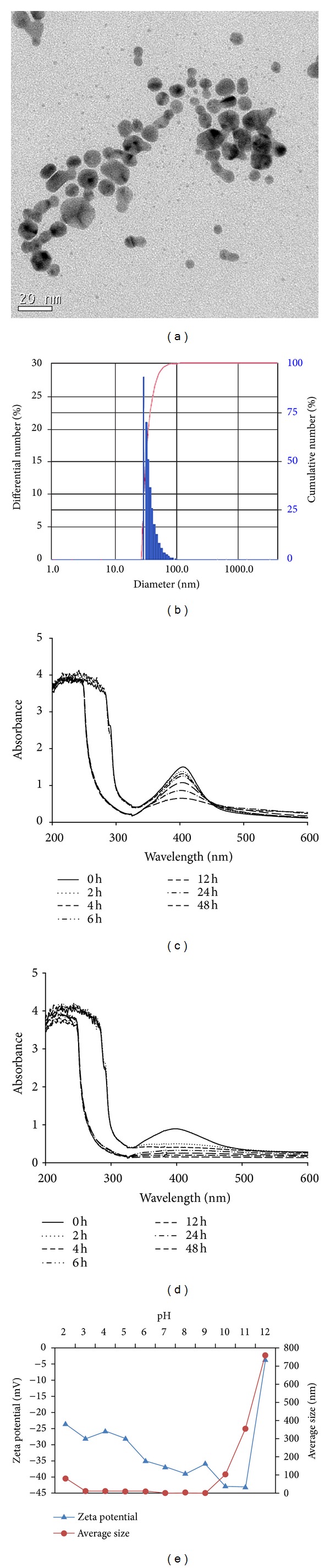
Physicochemical properties of AgNPs. (a) TEM image of AgNP prepared by dispersing the powder in deionized water. (b) Size distribution (number distribution) of AgNPs measured with ELS. (c) UV absorption spectrum of 10 mg/L AgNPs dispersed in DW. (d) UV absorption spectrum of 10 mg/L AgNPs dispersed in culture water. (e) pH-dependent variations of hydrodynamic sizes and zeta potential of AgNPs.

A representative TEM image of AgNPs is shown in Figure 1(a). The AgNPs employed in this study had a slightly elliptical or multifaceted shape with 8.30 ± 4.35 nm in size, although a few large particles were present. As prepared for stock suspension, AgNPs were characterized using the DLS method. The calculated size distribution histogram revealed that the size of AgNPs was 36.8 ± 10.2 nm (Figure 1(b)). The maximum absorbance spectra were around 410 and 400 nm in DW and culture water, respectively, (Figures 1(c) and 1(d)). The measured diameter of AgNPs only slightly changed within a pH range of 3–9, (Figure 1(e)). These data indicate that AgNPs exhibit a homogeneous dispersion in aqueous solutions.

Table S1 (see Table  S1 and Figure  S1 in Supplementary Material available online at http://dx.doi.org/10.1155/2013/494671) showed the acute toxicity of AgNP to adult and embryo medaka. LC50 values after 96 h exposure were calculated as 0.84 mg/L (CI: 0.67–1.00). In comparison, the acute toxicity level for adults was 0.80 mg/L (CI: 0.65–0.96). The present study demonstrates that the AgNPs used here were acutely lethal to both embryo and adult medaka. Many studies have reported variable acute toxicity levels of AgNPs on teleosts, with LC50s ranging from 0.0346 to 250 mg/L, depending on species and exposure duration (Table 1). Previously, the 96 h AgNP LC50 value was reported to be 0.0346 mg/L in medaka exposed to AgNPs with a size of 49.6 nm in suspension [20]. In another study on medaka, Wu et al. [24] reported that the 48 h LC50 value for 20–37 nm AgNPs was 1.03 mg/L, which was higher than the value found by Chae et al. [20], indicating lower toxicity. In *Danio rerio*, the LC50 value was 250 mg/L (24 h exposure, undefined) [26], 0.084 mg/L (48 h exposure, 0.2% polyvinyl pyrrolidone (PVP)-coated, 73.55 nm) [25], and 7.07 mg/L (48 h exposure, uncoated, 44.5 and 216 nm) [17]. The 96 h LC50 values for *Pimephales promelas* were 0.0894 and 0.0461 mg/L, respectively, for 10 nm uncoated AgNP [29]. For embryos, the 96 h LC50 value for *P. promelas* was 10.6 mg/L for ≤100 nm uncoated AgNPs [13], which was far less toxic than that found for medaka (LC50 value = 1.39 mg/L, uncoated, 3.6 nm nominal size) by Kashiwada et al. [7] and the current study. For *D. rerio *fry, 96 h LC50s of uncoated AgNP, monodispersed AgNP, and PVP-AgNP were 0.210, 0.088, and 0.162 mg/L, respectively [30]. In another study on *D. rerio*, the LC50 values for AgNPs of 3, 10, 50, and 100 nm were suggested 10.07, 13.55, 13.69, and 14.81 mg/L, respectively [31]. These results indicate that AgNPs with different sizes and coating agent conditions (i.e., with and without coating) could deliver different degrees of toxicity for different developmental stages of teleosts under different exposure times. To date, however, few studies have produced definitive evidence on why this is so, and on the mechanism of toxicity.

While the toxicity pattern of AgNPs to embryos dramatically increased during 96 h exposure, a rapid increase in toxicity occurred at 24 h exposure in adults. The LC50s of the embryo test after 24 to 96 h exposure decreased from 1.46 mg/L (CI: 0.81–2.10) to 0.84 mg/L (CI: 0.67–1.00). In comparison, the 24 h LC50 value in the acute adult toxicity test was not calculated (>1 mg/L), with no change in LC50s over the 48 h exposure period (0.8 mg/L; CI: 0.68–0.96). These observations may be attributed to differences in medaka's developmental stage and the aggregation characteristics of AgNPs. It has been generally suggested that fish embryos are less affected by waterborne chemicals than larvae or adults, because of the protective barrier formed by the chorion [32–35]. In a study using transparent medaka, adult medaka mainly accumulated 39.4 nm NPs in the gills and intestine [36]. In the current study, AgNPs showed fast aggregation in culture water within 2 h (Figure 1(d)). Although the extent of AgNP accumulation was not determined, the chorion may delay AgNP exposure to embryos, indicating that exposure characteristics regulate the time interval of toxicity occurrence between embryo and adult stages. In addition, considering the fast aggregation of AgNPs, the toxicity pattern in adult medaka was due to the uptake of AgNPs at the onset of exposure.

LC50s values from the embryonic lethal test dramatically decreased from 1.46 mg/L (CI: 0.81–2.10) to 0.43 mg/L (CI: 0.37–0.49) at 8 d after AgNP exposure when embryos started to hatch. Previous studies have reported the transport characteristics of AgNPs into the chorion layers of the embryos of various teleosts, including *O. latipes*, *D. rerio*, and *P. promelas* [13, 19, 37]. Kashiwada [37] reported that particles with 39.4–42,000 nm in diameter were adsorbed in the chorion of medaka eggs and accumulated in the oil droplets. In particular, particles of 39.4 nm in diameter shifted into the yolk and gallbladder during embryonic development. In the experiment using a fluorescent probe, AgNPs (5–46 nm) have been shown to enter zebrafish chorion pores via diffusion, indicating that NPs dock onto the chorion pore canals [19]. These trapped AgNPs might cause severe damage during embryonic development by having the chorion pore canals blocked, which might affect membrane transport, and consequently destroy the integrity of the egg chorion, causing premature hatching [19]. The results of the hatching rate and body length assessments supported this hypothesis (Figure 4). AgNPs caused a dose-dependent decrease in the hatching rate and body length of fry medaka. The hatching rate was reduced by up to 13.3% at the second highest exposure level compared to the control, and 100% mortality occurred at the highest concentration (1 mg/L). 

**Figure 2:**
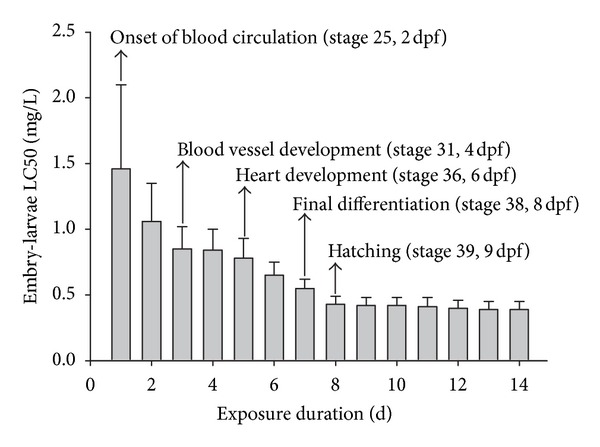
LC50s values for *Oryzias latipes* embryos and larvae exposed to different AgNPs concentrations for 14 d. Error bars indicate the upper range of the 95% confidence interval. The box indicates the stages at which the heart rate of *Oryzias latipes* was measured. “dfp” indicates postfertilization.

**Figure 3:**
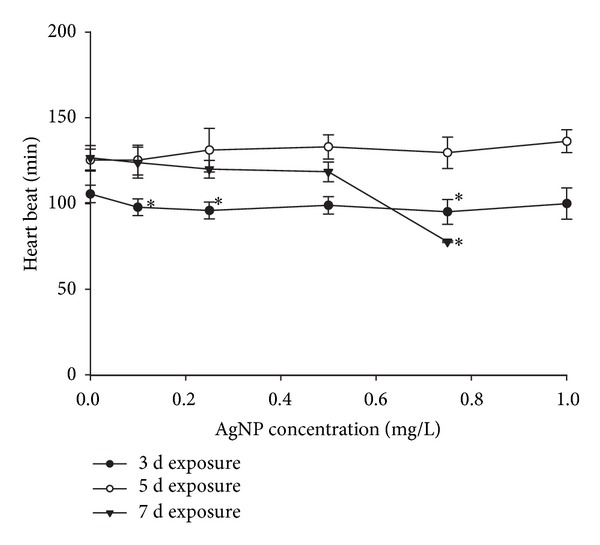
Heart rate of *Oryzias latipes* embryos after 3, 5, 7, and d exposure to 0, 0.1, 0.25, 0.5, 0.75, and 1 mg/L AgNPs. Asterisk indicates a significant difference from the control (*P < 0.05) based on Dunnett's analysis of variance. Values are presented as mean ± standard deviation.

**Figure 4:**
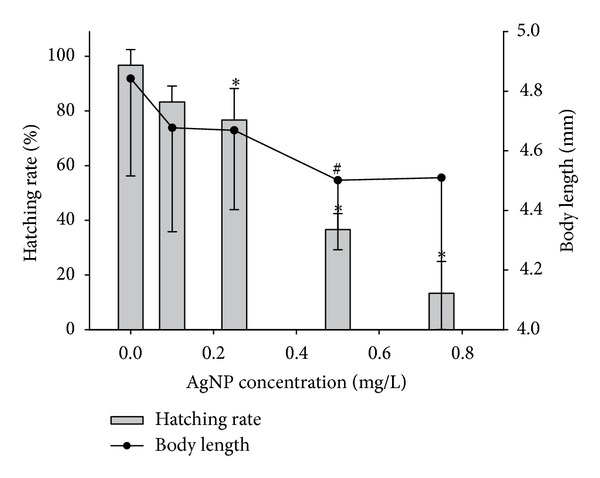
Hatching rate and body length of *Oryzias latipes* embryos and larvae after 14 d exposure to different concentrations of AgNPs (0, 0.1, 0.25, 0.5, 0.75, and 1 mg/L). Asterisk and number signs indicate a significant difference from the control (^∗,#^ *P* < 0.05) based on Dunnett's analysis of variance. Values are presented as mean ± standard deviation.

In addition to differences in the toxicity pattern between embryo and adult medaka, we observed an interesting stepwise increase in toxicity that was dependent on the developmental stage of the embryo (Figure 2). Lethal embryonic toxicity increased during the first (from exposure day 1 to 3) and last (from exposure day 5 to 8) periods of embryonic development, whereas there was no change during the middle period (from exposure day 3 to 5). In the experiment assessing the vulnerable periods for developmental ethanol toxicity to medaka embryos, Oxendine et al. [38] concluded that sensitivity to ethanol toxicity varies according to critical periods. Developmental stage-dependent sensitivity was also reported by González-Doncel et al. [39]. This result provides some insights into understanding our observation. Vulnerable periods for developmental AgNP toxicity started at stage 25 of onset of blood circulation (2 d postfertilization (dpf)), and at stage 36 of heart development (6 dpf) in the present study (Figure 2). Wu et al. [24] reported that AgNP exposure from stage 10 to 2 dpf (from exposure day 0 to 1 in the present study) induced various deformities in morphogenesis (particularly, sluggish circulation and hemorrhage), with such severe circulatory abnormalities being associated with the occurrence of pericardial edema and tube heart [24]. We also found that 7 d exposure to 0.8 mg/L of AgNP at the end of the second vulnerable period of embryonic development resulted in significant heart beat retardation in medaka embryos (Figure 3). However, it is not sure whether developmental vulnerability to AgNP toxicity is caused by hemorrhaging or heart edema, as tests have not been conducted to determine hemorrhaging in embryos.

Gottschalk et al. [40] modeled AgNPs concentration using the probabilistic material flow analysis and calculated range from 0.116 to 0.764 ng/L in the surface water. In another study, the value of predicted environmental concentration was 0.03 *μ*g/L [41]. The exposure level (0.1–1 mg/L of AgNPs) applied in this study was much higher than that in surface water. To evaluate realistically the effect of AgNPs, it is necessary to assess toxicity test with environmentally relevant concentration. 

## 4. Conclusion

Only a few reports on AgNP toxicity have been published to date, despite the limited understanding about the unintended toxicity of AgNPs to aquatic organisms and its mechanism of contamination. In the present study, we provided experimental evidence regarding developmental stage-dependent sensitivity to AgNPs, as well as information about the different characteristics of toxicity between embryo and adult medaka. 

## Supplementary Material

The cumulative survival rate and LC50 values of *Oryzias latipes* embryo on exposure to different AgNPs concentrations over a 14-d period were presented in Fig. S1 and Table S1, respectively. To compare sensitivity between embryo and adult, LC50s of adult medaka for 24h ~ 96h were also suggested (Table S1).

## Figures and Tables

**Table 1:** Acute lethal toxicity of AgNPs to adults and embryos of freshwater teleosts.

Species (age or stage)	Exposure duration (h)	LC50 (95% CI)^1^ (mg/L)	Reference
*Oryzias latipes* (~4 month old)	96	0.80 (0.65–0.96)	This study
*O. latipes* (adult)	48	1.03	[24]
*O. latipes* (adult)	96	0.0346	[20]
*Danio rerio* (adult)	48	0.084	[25]
*D. rerio* (adult)	48	7.07 (6.04–8.28)	[17]
*D. rerio* (adult)	24	250	[26]
*Oncorhynchus mykiss* (adult)	96	2.3	[27]
*Hypophthalmichthys molitrix* (adult)	96	66.4	[28]
*Carassius auratus *(adult)	96	83.9	[28]

*O. latipes* (embryo, <24 h of spawning)	96	0.84 (0.67–1.00)	This study
*O. latipes* (embryo, <24 h of spawning)	96	1.39	[7]
*Pimephales promelas *(embryo, <24 h of spawning)	96	10.6	[13]

^1^95% confidence intervals.
