# Supplementary material for: Stepwise Embryonic Toxicity of Silver Nanoparticles on Oryzias latipes
Source: Biomed Res Int. 2013 Jul 30;2013:494671. doi: 10.1155/2013/494671 (PMC3745929; doi:10.1155/2013/494671)
Supplement: Supplementary file 1 — The cumulative survival rate and LC50 values of Oryzias latipes embryo on exposure to different AgNPs concentrations over a 14-d period were presented in Fig. S1 and Table S1, respectively. To compare sensitivity between embryo and adult, LC50s of adult medaka for 24h ~ 96h were also suggested (Table S1). [file 494671.f1.pdf]

Supporting information

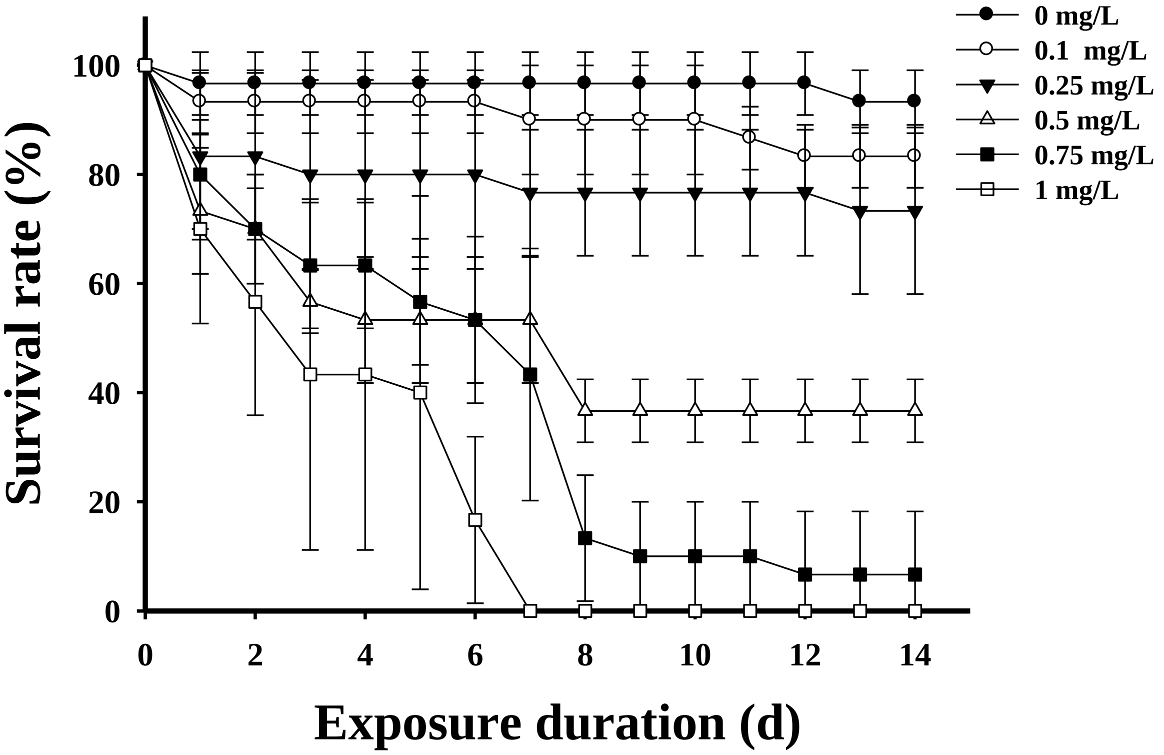

Fig. S1. The cumulative survival rate of *Oryzias latipes* embryos on exposure to different AgNPs concentrations over a 14-d period.

Table S1. Acute toxicity of AgNPs to embryos of *Oryzias latipes*

Exposure duration	LC50 (95% confidence interval) (mg/L)	
	Embryo (<24 h of spawning)	Adult (~4 months old)
24 h	1.46 (0.81-2.10)	>1
48 h	1.06 (0.78-1.35)	0.80 (0.65-0.96)
74 h	0.85 (0.68-1.02)	0.80 (0.65-0.96)
96 h	0.84 (0.67-1.00)	0.80 (0.65-0.96)
5 d	0.78 (0.64-0.93)	
6 d	0.65 (0.56-0.75)	
7 d	0.55 (0.47-0.62)	
8 d	0.43 (0.37-0.49)	
9 d	0.42 (0.36-0.48)	
10 d	0.42 (0.36-0.48)	
11 d	0.41 (0.35-0.48)	
12 d	0.40 (0.34-0.46)	
13 d	0.39 (0.32-0.45)	
14 d	0.39 (0.32-0.45)	
